# Supplementary figures and images for: Bifurcations and bursting in the Epileptor
Source: PLoS Comput Biol. 2024 Mar 6;20(3):e1011903. doi: 10.1371/journal.pcbi.1011903 (PMC10947678; doi:10.1371/journal.pcbi.1011903)

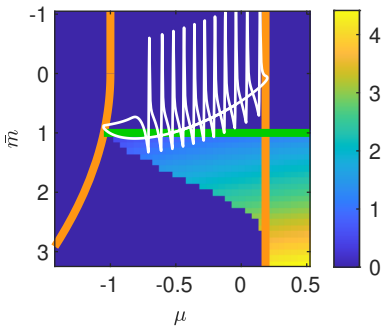

Supplement: S1 Folder — The folder contains the Matlab code used to generate the figures. (ZIP) [file pcbi.1011903.s001.zip › SaggioJirsa_2024PlosCB_code/Fig6/Fig_6_map2.pdf]

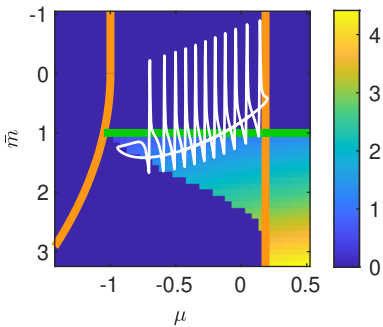

Supplement: S1 Folder — The folder contains the Matlab code used to generate the figures. (ZIP) [file pcbi.1011903.s001.zip › SaggioJirsa_2024PlosCB_code/Fig6/Fig_6_map1.pdf]

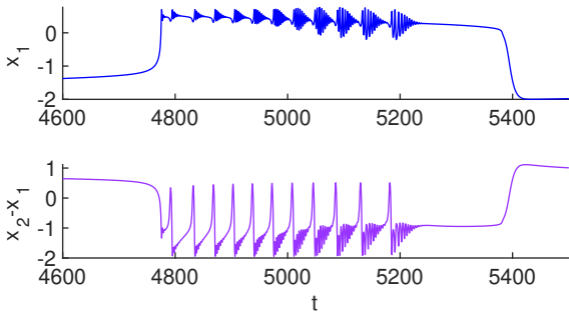

Supplement: S1 Folder — The folder contains the Matlab code used to generate the figures. (ZIP) [file pcbi.1011903.s001.zip › SaggioJirsa_2024PlosCB_code/Fig6/Fig_6_timeseries2.pdf]

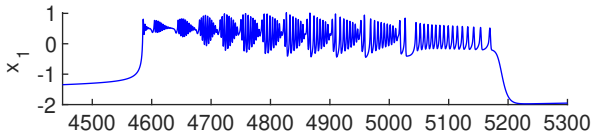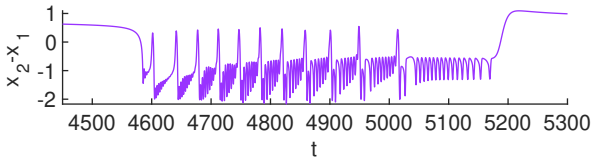

Supplement: S1 Folder — The folder contains the Matlab code used to generate the figures. (ZIP) [file pcbi.1011903.s001.zip › SaggioJirsa_2024PlosCB_code/Fig6/Fig_6_timeseries1.pdf]

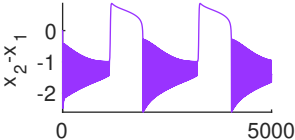

Supplement: S1 Folder — The folder contains the Matlab code used to generate the figures. (ZIP) [file pcbi.1011903.s001.zip › SaggioJirsa_2024PlosCB_code/Fig7/Fig_x0_timeseries2_1.pdf]

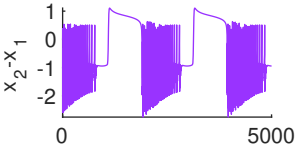

Supplement: S1 Folder — The folder contains the Matlab code used to generate the figures. (ZIP) [file pcbi.1011903.s001.zip › SaggioJirsa_2024PlosCB_code/Fig7/Fig_x0_timeseries2_2.pdf]

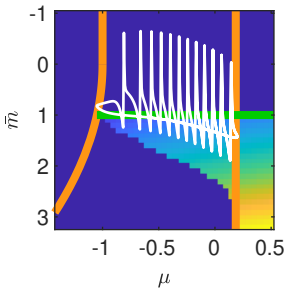

Supplement: S1 Folder — The folder contains the Matlab code used to generate the figures. (ZIP) [file pcbi.1011903.s001.zip › SaggioJirsa_2024PlosCB_code/Fig7/Fig_x0_map_1.pdfFig_x0_map_2.pdf]

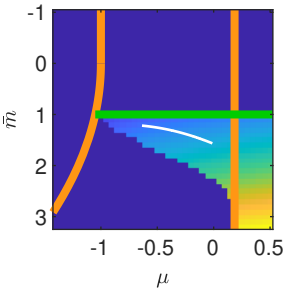

Supplement: S1 Folder — The folder contains the Matlab code used to generate the figures. (ZIP) [file pcbi.1011903.s001.zip › SaggioJirsa_2024PlosCB_code/Fig7/Fig_x0_map_3.pdfFig_x0_map_1.pdf]

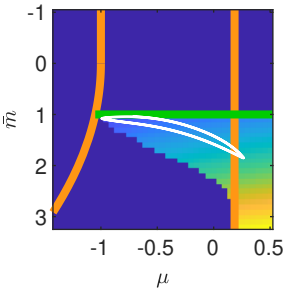

Supplement: S1 Folder — The folder contains the Matlab code used to generate the figures. (ZIP) [file pcbi.1011903.s001.zip › SaggioJirsa_2024PlosCB_code/Fig7/Fig_x0_map_2.pdfFig_x0_map_1.pdf]

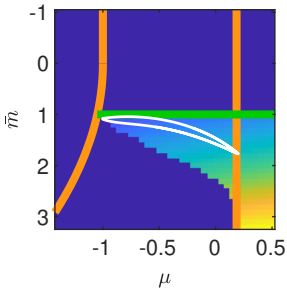

Supplement: S1 Folder — The folder contains the Matlab code used to generate the figures. (ZIP) [file pcbi.1011903.s001.zip › SaggioJirsa_2024PlosCB_code/Fig7/Fig_x0_map_1.pdfFig_x0_map_1.pdf]

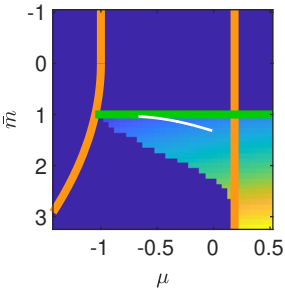

Supplement: S1 Folder — The folder contains the Matlab code used to generate the figures. (ZIP) [file pcbi.1011903.s001.zip › SaggioJirsa_2024PlosCB_code/Fig7/Fig_x0_map_3.pdfFig_x0_map_2.pdf]

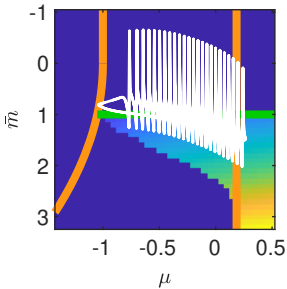

Supplement: S1 Folder — The folder contains the Matlab code used to generate the figures. (ZIP) [file pcbi.1011903.s001.zip › SaggioJirsa_2024PlosCB_code/Fig7/Fig_x0_map_2.pdfFig_x0_map_2.pdf]

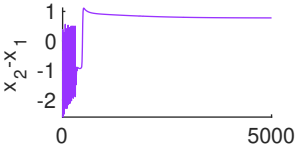

Supplement: S1 Folder — The folder contains the Matlab code used to generate the figures. (ZIP) [file pcbi.1011903.s001.zip › SaggioJirsa_2024PlosCB_code/Fig7/Fig_x0_timeseries3_2.pdf]

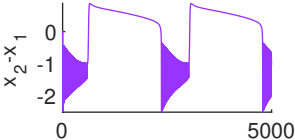

Supplement: S1 Folder — The folder contains the Matlab code used to generate the figures. (ZIP) [file pcbi.1011903.s001.zip › SaggioJirsa_2024PlosCB_code/Fig7/Fig_x0_timeseries1_1.pdf]

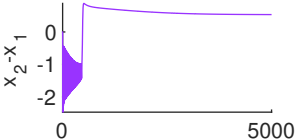

Supplement: S1 Folder — The folder contains the Matlab code used to generate the figures. (ZIP) [file pcbi.1011903.s001.zip › SaggioJirsa_2024PlosCB_code/Fig7/Fig_x0_timeseries3_1.pdf]

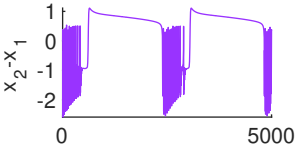

Supplement: S1 Folder — The folder contains the Matlab code used to generate the figures. (ZIP) [file pcbi.1011903.s001.zip › SaggioJirsa_2024PlosCB_code/Fig7/Fig_x0_timeseries1_2.pdf]

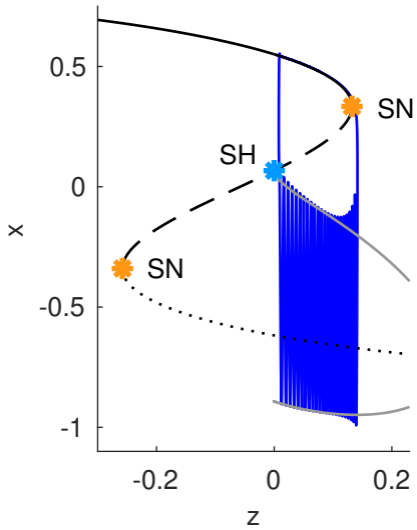

Supplement: S1 Folder — The folder contains the Matlab code used to generate the figures. (ZIP) [file pcbi.1011903.s001.zip › SaggioJirsa_2024PlosCB_code/Fig1B/SN_SH_bursting.pdf]

# Amplitude

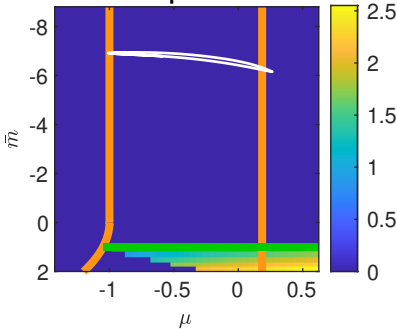

Supplement: S1 Folder — The folder contains the Matlab code used to generate the figures. (ZIP) [file pcbi.1011903.s001.zip › SaggioJirsa_2024PlosCB_code/Fig5/Fig5_map4.pdf]

# Frequency

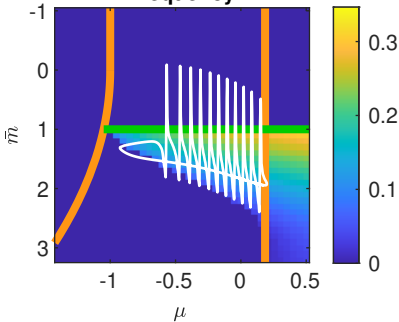

Supplement: S1 Folder — The folder contains the Matlab code used to generate the figures. (ZIP) [file pcbi.1011903.s001.zip › SaggioJirsa_2024PlosCB_code/Fig5/Fig5_map2.pdf]

# Amplitude

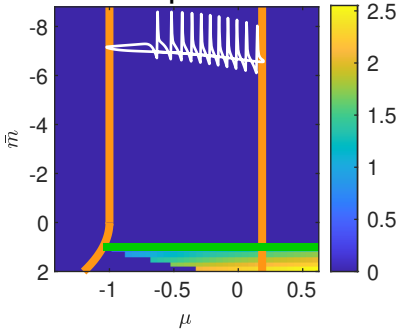

Supplement: S1 Folder — The folder contains the Matlab code used to generate the figures. (ZIP) [file pcbi.1011903.s001.zip › SaggioJirsa_2024PlosCB_code/Fig5/Fig5_map3.pdf]

# Amplitude

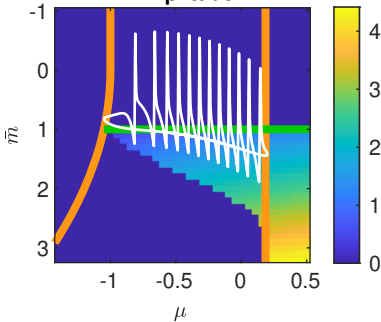

Supplement: S1 Folder — The folder contains the Matlab code used to generate the figures. (ZIP) [file pcbi.1011903.s001.zip › SaggioJirsa_2024PlosCB_code/Fig5/Fig5_map1.pdf]

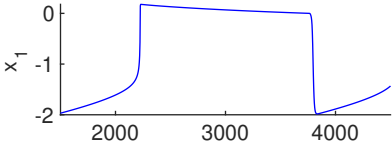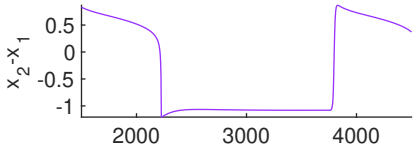

Supplement: S1 Folder — The folder contains the Matlab code used to generate the figures. (ZIP) [file pcbi.1011903.s001.zip › SaggioJirsa_2024PlosCB_code/Fig5/Fig5_timeseries4.pdf]

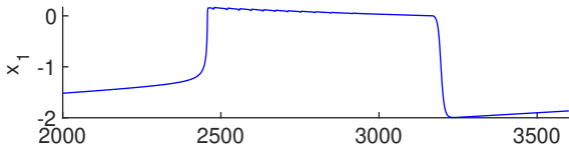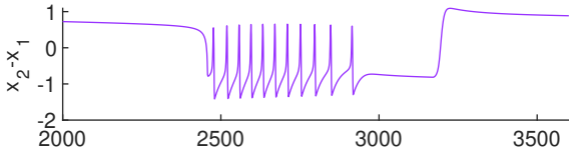

Supplement: S1 Folder — The folder contains the Matlab code used to generate the figures. (ZIP) [file pcbi.1011903.s001.zip › SaggioJirsa_2024PlosCB_code/Fig5/Fig5_timeseries3.pdf]

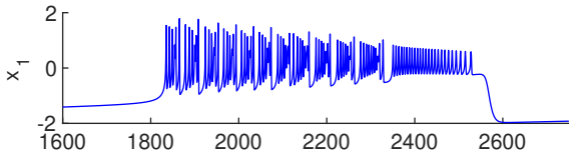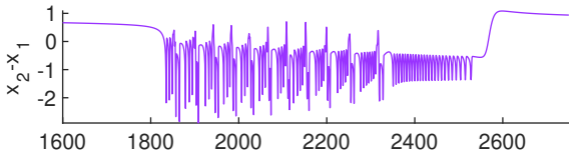

Supplement: S1 Folder — The folder contains the Matlab code used to generate the figures. (ZIP) [file pcbi.1011903.s001.zip › SaggioJirsa_2024PlosCB_code/Fig5/Fig5_timeseries2.pdf]

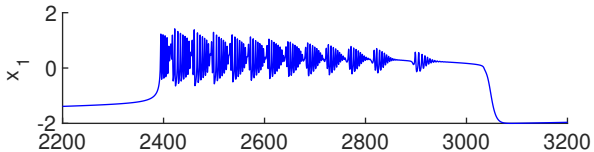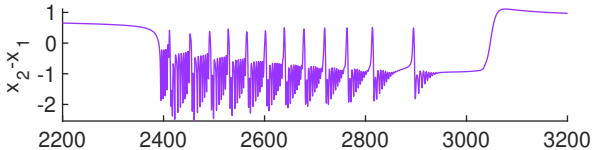

Supplement: S1 Folder — The folder contains the Matlab code used to generate the figures. (ZIP) [file pcbi.1011903.s001.zip › SaggioJirsa_2024PlosCB_code/Fig5/Fig5_timeseries1.pdf]

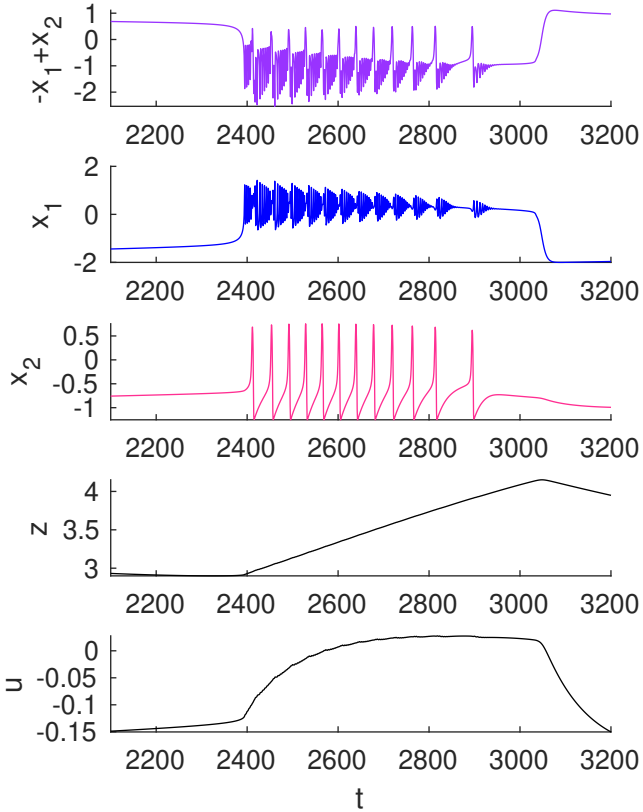

Supplement: S1 Folder — The folder contains the Matlab code used to generate the figures. (ZIP) [file pcbi.1011903.s001.zip › SaggioJirsa_2024PlosCB_code/Fig2/Epileptor.pdf]

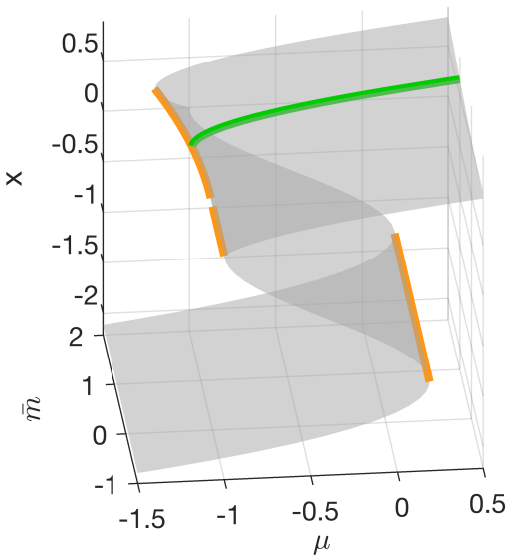

Supplement: S1 Folder — The folder contains the Matlab code used to generate the figures. (ZIP) [file pcbi.1011903.s001.zip › SaggioJirsa_2024PlosCB_code/Fig3/Fig3A_FixedPoints_manifold.pdf]

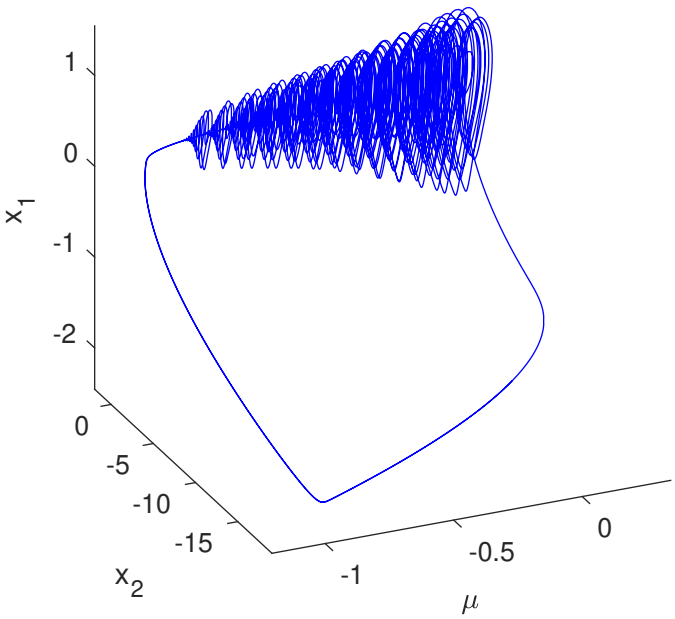

Supplement: S1 Folder — The folder contains the Matlab code used to generate the figures. (ZIP) [file pcbi.1011903.s001.zip › SaggioJirsa_2024PlosCB_code/Fig3/Fig3B_trajectory.pdf]

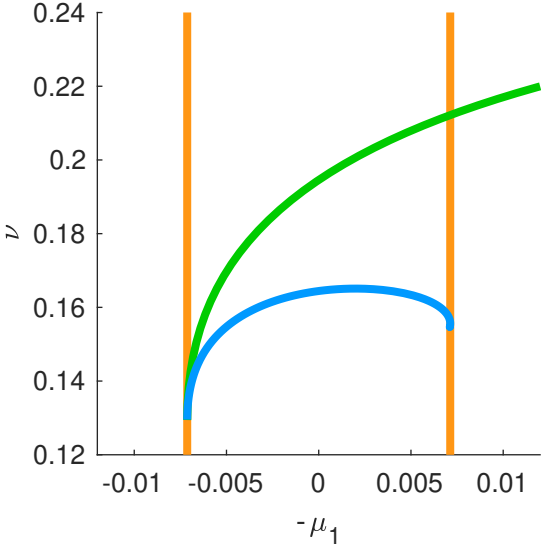

Supplement: S1 Folder — The folder contains the Matlab code used to generate the figures. (ZIP) [file pcbi.1011903.s001.zip › SaggioJirsa_2024PlosCB_code/Fig1A/map_DTB.pdf]
