# Supplementary material for: Bifurcations and bursting in the Epileptor
Source: PLoS Comput Biol. 2024 Mar 6;20(3):e1011903. doi: 10.1371/journal.pcbi.1011903 (PMC10947678; doi:10.1371/journal.pcbi.1011903)
Supplement: Attachment — Submitted filename: Response_to_reviewers.pdf [file pcbi.1011903.s002.pdf]

Dear Dr. Taylor and Dr. Serre,

Thank you for your fast feedback about our manuscript.

We prepared a new version of the draft to address the concerns raised by the reviewers. Since there were no issues reported with the Results and Methods sections, we focused on reworking the Introduction and the Discussion.

The main issues raised by the reviewers concerned:

• *Usefulness of phenomenological models and large-scale brain models to improve surgery outcome in epilepsy* (R1) - We give a better introduction to this approach and the current literature. We also make clearer why, given the goal of being applied in the context of surgery, these models aim at reproducing features of focal seizures and not generalized ones (R1).

• *Usefulness of classifying seizures based on dynamics (R1) and relationship with an existing classification* (R2) - We clarify why we think that classifying seizures based on the underlying dynamics can usefully complement current classifications and briefly discuss how these taxonomies relate to other existing ones.

• *Discussion of other dynamical mechanisms for seizure onset beyond bifurcations* (R1 & R2) - We discuss other dynamical mechanisms, beyond bifurcations, that have been proposed to describe seizure onset focusing on those found in models used for large-scale personalized brain modeling, and discuss how the Epileptor stands with regards to these other mechanisms.

We also addressed all the other points as detailed in the response to the reviewers.

We thank the reviewers for helping us clarify the framework in which this work is placed and to elaborate better on some of our assumptions and results.

As a note, this work does not aim at introducing new classifications nor models, rather the goal is to give a thorough, and we hope useful, description of a model that is already being used by several researchers. While we acknowledge that an improved contextualisation of the framework and results greatly improves our manuscript, we tried to keep it concise and to refer the interested reader to the specific papers (Jirsa et al. 2014 for the Epileptor, Saggio et al. 2020 for the taxonomy and Wang et al. 2023 for a review on the use of Brain Twins in epilepsy) where we go in more details on these specific topics.

We hope that the current version of the manuscript, together with the rebuttal letter, will clear the concerns raised and that our work can be judged suitable for publication.

We would also like to change the title of our manuscript to ‘Bifurcations and bursting in the Epileptor’ to better reflect the content.

Sincerely,

Maria Luisa Saggio,
Viktor Jirsa

***

Please find below our detailed responses to the raised concerns.

Reviewer #1: The paper considers the bifurcation analysis in a some neuron model (theoretical papers in a very narrow field), but pretends to improve surgical outcomes (the great practical problem). Unfortunately, the authors ignored all the progress made in investigation of epilepsy till now. It is hard to express all the problems in a single review, but I will try to mark at least some main points.

The model we are dealing with is not a neuron model, rather a neural mass model, we clarified that in the introduction saying that it is used to model a brain region.

Despite the field being recent, the use of large-scale brain models incorporating the dynamics of neural mass models is rapidly growing and showing promising initial results that could potentially have an impact in the clinics. In particular, one of these large-scale models, the Virtual Epileptic Patient, is currently under clinical trial in France as the EPINOV project (Improving Epilepsy surgery management and progNOsis using Virtual brain technology) involving 10 hospitals and more than 300 patients. Given that the VEP model makes use of the Epileptor model, we believe a deeper understanding of this model behaviors is necessary for at least two reasons: (i) have a better control over its dynamics and (ii) simulate other dynamical classes of seizures (see reply to the next point) to verify whether this could improve patient specificity. These are the reasons that have motivated us to produce the present work. We have modified the text to make our goal clearer. We have also added in the discussion that the impact of simulating the VEP model with different classes is unknown and remains to be assessed.

In reply to this point we have added/modified the following paragraphs:

‘In addition, due to their low computational burden, phenomenological models for seizure activity are prime candidates to be used in whole-brain simulations. Such patient- specific models incorporate patient data, such as structural or functional data, to build large scale networks in which brain regions are endowed with a dynamical model and coupled together. Most studies using this approach in the context of epilepsy have focused on potential strategies to improve surgical outcome in drug-resistant patients. For some of these patients, an alternative to medication is the surgical resection of the brain regions involved in the generation of seizures, the epileptogenic zone (EZ), under the constraint of limiting post-surgical neurological impairments [Bartolomei and Wendling, 2009]. However, outcomes of this type of surgery are very variable and depend on the patient's condition and epilepsy, with surgery success rates between 34% and 74% [Jobst and Cascino, 2015]. The inference of the EZ is highly non trivial and large-scale patient specific approaches aim at arming clinicians with an additional tool to use in their evaluation. This tool leverages on the possibility, through whole brain simulations, to reveal otherwise hidden complex networks and dynamical effects. It can be used to test specific clinical hypotheses (e.g. ‘will seizures stop by removing a specific set of brain regions?’) by simulating functional data or to find unbiased optimal surgery strategies through the applications of different metrics or parameters fitting techniques. These models can con- tribute to different stages of the presurgical evaluation by studying seizure propagation, resection strategies, predicted surgery outcome or strategies to limit cognitive impairment [An et al., 2019; Gerster et al., 2021; Goodfellow et al., 2016; Hutchings et al., 2015; Laiou et al., 2019; Lopes et al., 2017; Mill´an et al., 2022; Olmi et al., 2019; Proix et al., 2017; 2018; Sinha et al., 2016; Sip et al., 2021; Taylor et al., 2014; Vattikonda et al., 2021; Wang et al., 2023; Yang et al., 2018], or to improve presurgical implantation of intracranial electrodes [Lopes et al., 2020]. Other proposed applications, beyond surgery, are related to the diagnosis of focal and generalized epilepsy [Lopes et al., 2019], the eval\uation of the increased proneness to seizures in Alzheimer’s disease [Tait et al., 2021] and the investigation of the effects of brain stimulation [Giannakakis et al., 2020].

The initial results from these retrospective studies have been promising, and one of these frameworks, the Virtual Epileptic Patient (VEP) [Jirsa et al., 2017; Proix et al., 2017; Wang et al., 2023], is currently being validated in a prospective clinical trial involving more than 300 patients in France. Patient-specificity is key and, under the requirement that state of the art methods are used in diffusion imaging, it has been shown that the patient- specific connectome gives the best outcome for the VEP [Proix et al., 2017]. At the brain region level, the Epilepor model is

used for all patients. This model has been proposed by Jirsa et al. [2014] to phenomenologically reproduce the most predominant dynamotype as observed in in vitro preparations, zebrafish, mice and human epileptic patients with focal seizures. These patients, though, exhibited other dynamotypes as well [Jirsa et al., 2014; Saggio et al., 2020]. The impact of the dynamotype on the VEP outcome has not yet been investigated, but we know that different classes may behave differently in terms of synchronization and propagation properties [Belykh et al., 2005; Izhikevich, 2007]. Exploring the full potential of the Epileptor model in terms of bursting dynamics, and how to set the parameters in order to obtain different classes, is an important step to (i) improve our understanding of the model (ii) explore the possibility that a further personalization of the VEP model in term of patient-specific dynamotype could alter the model outcome.'

1. There are 30-40 types of epilepsy following ILAE. These types are actually different diseases. Each of them has its own brain structures involved, e.g. for absence epilepsy thalamocortical system is involved, though for the limbic one hippocampus and parahippocampal areas play the main role. Each of them has its own mechanism of seizure initiation, maintenance and termination. Each of them is characterized by signals with specific type, spectrum and seizure duration. Nothing of this was accounted in the paper. All the progress done was ignored. Therefore, I should conclude that the authors are compeltely out of topic and their text is not professional.

We thank the reviewer for having raised this point, which we care much about. In fact, we are well aware that epilepsy is a complex multifactorial disease with multiple etiologies, structures and networks involved, biophysical mechanisms for seizure initiation, evolution and termination, multiple clinical phenomenology, and so on. What we find striking is that, despite all these different routes to epilepsy and diverse biophysical substrate, electrographic seizures are highly stereotypical among patients, and even across species and small neuronal networks (Jirsa et al. 2014), as we explain in the introduction. This is exactly the reason that lead to the proposal that, if different causes and biophysical mechanisms can concur to generate similar dynamics, and if epilepsy is essentially a dynamical disease (Glass and Mackey 1988, Andrzejak et al. 2001, 2003, Lopes da Silva et al. 1994, 2002, 2003), it is worth understanding and classifying these 'final' dynamics per se. This is because different dynamical systems behave differently (e.g. type of stimulation needed to stop oscillations, synchronization and propagation properties) and this

can impact treatment even if brought about by diverse etiology or biophysical mechanisms. This is why us (Jirsa et al. 2014), and other authors (Wang et al. 2014) proposed that a classification of seizures based on dynamics could complement current classifications. The taxonomy we talk about in the manuscript is based on onset/offset bifurcation pairs (Jirsa et al. 2014, Saggio et al. 2020), but we think this is one of the relevant dynamical features and that the taxonomy should be enriched with any kind of informations on the underlying dynamics mechanisms (Saggio et al. 2023), such as dynamical mechanisms for transitions (bifurcation, noise-induced, intermittency…) as pointed out by the reviewers, and so on.

In reply to this point we have added/modified the following paragraphs:

‘This translates in the striking fact that, despite the described heterogeneity, seizures’ electrographic signatures are remarkably stereotyped across patients [Perucca et al., 2013], different animal species and primitive laboratory models [Jirsa et al., 2014]. This limited variability, together with epilepsies being dynamical diseases characterized by abnormal dynamics [Andrzejak et al., 2001; Milton, 2000; Silva et al., 2003; 1994], has been pro- posed to reflect the existence of invariant dynamical properties in seizures’ underlying mechanisms that could result form a variety of biological processes.

Models that focus on reproducing relevant features observed in data, that is phenomenological models, are particular useful in the study of epileptic seizures, because they allow to focus on these essential dynamics without committing to a specific choice in terms of biological implementation. Insights from this class of models can complement but also guide our understanding of the dynamical repertoire of more complex biophysically inspired models, establishing a link between abstract dynamical and specific physiological mechanisms (Depannemaecker et al. [2022]).

The identification of a series of abstract dynamical mechanisms characterizing seizures allows to add a new dimension to current clinical classification that is operational and based on phenotype [Fisher et al., 2017]. There are a few initial proposals in this direc- tion. Some exploits more detailed spatiotemporal dynamics. For example, spatiotemporal heterogeneities in the distribution of excitability within a single brain region produce either low amplitude fast activity (LAF) or high amplitude slow activity (HAS) at seizure on- set through different

dynamical mechanisms that correlate with surgical outcomes [Wang et al., 2014]. Spatiotemporal dynamics at different scales also provides dynamical mechanisms underlying synchronous or asynchronous seizure termination across brain regions [Proix et al., 2018]. Another, complementary, proposal neglects the spatial component within a brain region and assumes that the mesoscopic abrupt transitions between interictal and ictal states are brought about by bifurcations. Classes are defined as the onset/offset bifurcations pair that delimitates the seizure [Jirsa et al., 2014], extending to epilepsy a taxonomy that was previously developed by Izhikevich [2000] for use in neuronal bursting. In the context of epilepsy, we call these bursting classes 'dynamotypes' [Saggio et al., 2020], establishing the taxonomy we refer to in the present work.'

'In this work, we refer to the taxonomy of seizures based on dynamics, where a class is defined by its onset/offset bifurcation pair. This framework underpins the development of the Epileptor. However, other information that contributes to identifying patient-specific or seizure-specific dynamical mechanisms can also enhance the dynamic classification of seizures. These include different onset mechanisms at the neural mass level, as previously discussed, and more fine-grained spatiotemporal descriptions required for understanding the mechanisms of seizure onset, evolution, and termination [Proix et al., 2018; Wang et al., 2014]. These taxonomies may be orthogonal to other classification systems, patholo- gies, or localizations [Saggio et al., 2020]. However, the importance of modeling various seizures' dynamical features may vary depending on the focus of a specific investiga- tion. For example, the intermediate subsystem of the Epileptor enhances the similarity between certain dynamotypes and observed recordings. Specifically, Perucca's class of low- frequency, high-amplitude periodic spikes (ii) [Perucca et al., 2013] seems well-represented by the Epileptor's classes with a SupH onset, as shown in 6. This subsystem is also crucial for understanding the rapid spread of ictal waves in the form of spike-and-wave complexes and synchronous seizure termination across brain regions [Proix et al., 2018]. However, its role is less pronounced in examining seizure propagation across different brain regions [Proix et al., 2018], leading to its exclusion from the VEP pipeline in the EPINOV clinical trial for the estimation of the epileptogenic zone (EZ) network [Wang et al., 2023]. Nevertheless, during the trial with a large cohort, we observed that the data features not included might contain valuable insights. The impact of specific modeling choices on the performance of the VEP requires further characterization [Junges et al., 2019]. This study's findings suggest a potential approach in the VEP to systematically explore the relevance of

possible onset patterns, in terms of both noise-induced transitions and bifurcation, as well as specific bifurcation diagrams, in estimating EZ networks.'

2. The epilepsy modeling is a long story. Let me provide a small introduction to the absence epilepsy modeling since this form is the simplest one and very well studied.

I. When discussing the mechanisms of thalamocortical interactions, main results were achieved using two rat strains: WAG/Rij and GAERS. Please, consider the following papers (and many others can also be found):

1. Meeren H, Pijn J, van Luijtelaar G, Coenen A, Lopes da Silva F. Cortical focus drives widespread corticothalamic networks during spontaneous absence seizures in rats. Journal of Neuroscience. 2002;22:1480–1495. doi: 10.1523/JNEUROSCI.22-04-01480.2002

2. Coenen A and van Luijtelaar G. Genetic animal models for absence epilepsy: a review of the WAG/Rij strain of rats. Behavioral Genetics. 2003; 33(6): 635–655. doi: 10.1023/A:1026179013847

3. Lüttjohann A, van Luijtelaar G. The dynamics of cortico-thalamocortical interactions at the transition from pre-ictal to ictal LFPs in absence epilepsy. Neurobiology of Disease. 2012;47:47. 60.doi: 10.1016/j.nbd. 2012.03.023.

4. Sysoeva M, Lüttjohann A, van Luijtelaar G, Sysoev I. Dynamics of directional coupling underlying spike-wave discharges. Neuroscience. 2016;314:75–89. doi: 10.1016/j.neuroscience.2015.11.044.

5. Sysoeva M, Vinogradova L, Kuznetsova G, Sysoev I, van Rijn C. Changes in corticocortical and corticohippocampal network during absence seizures in WAG/Rij rats revealed with time varying Granger causality. Epilepsy and Behavior. 2016;64:44–50. doi: 10.1016/j.yebeh.2016.08.009.

6. Sysoeva M V, Sitnikova E, Sysoev I V. Thalamo-Cortical Mechanisms of Initiation, Maintenance and Termination of Spike-wave Discharges at WAG/Rijrats. Zhurnal vysshei nervnoi deiatelnosti imeni I P Pavlova. 2016;66(1):103-112. doi: 10.7868/S0044467716010123

II. There is a classical mean-field model [Suffczynski P, Kalitzin S, Lopes Da Silva FH. Dynamics of non-convulsive epileptic phenomena modeled by a bistable neuronal network. Neuroscience.2004; 126(2): 467-84. doi: 10.1016/j.neuroscience.2004.03.014] which explains the SWDs using a system of 26 equations (not all of them were written explicitly in the paper,

unfortunately, so you should reinvent some of them in order to use it), which is even more detailed than the proposed here model. It also includes GABA-ergic inhibition. If the authors model absence epilepsy, must indicate clearly what new the current study proposes in comparison to this model.

We thank the reviewer for these references. However, the Epileptor model was built to simulate focal seizures data from patients undergoing pre-surgical evaluation, so none of the patients had absence seizures and absence seizures are not the focus of our work. We now clarified this throughout the text.

Also, the model is phenomenological and agnostic to a specific biophysical implementation. This of course is a limitation of the model, if one aims at using it for goals that require a clearer mechanistic link to the biophysical substrate. On the other hand, it is advantageous when the goal is to isolate the essential dynamics and embed the model in large-scale networks to study seizure propagation where low computational cost is a plus. Of course, none of these approaches is better than the other and whether they are useful depends on the research question being asked. To clarify this point we improved on this paragraph in the revision:

‘Models that focus on reproducing relevant features observed in data, that is phenomenological models, are particularly useful in the study of epileptic seizures, because they allow to focus on these essential dynamics without committing to a specific choice in terms of biological implementation. Insights from this class of models can complement but also guide our understanding of the dynamical repertoire of more complex biophysically inspired models, establishing a link between abstract dynamical and specific physiological mechanisms (see for example Depannemaecker et al. [2022]).’

However, the history of generalized seizure modeling is of great importance for the development of the framework in which our work is placed, particularly because the first ‘unifying’ explanations for different types of seizures in one single model, and thus the concept of navigating the map, have been developed in this context (Breakspear et al. 2006, Lopes et al. 2003).

It is also interesting to know whether the Epileptor, even though not specifically designed to do so, can nevertheless produce phenomenological seizures compatible with ‘absence’ when the parameters are appropriately tuned, and in the present work we can rule out this possibility because all the classes we found have direct current shift that is instead absent in absence seizures. The DTB model instead allows for classes without DC shift that are compatible with modelization of absence seizures proposed in the literature. For example: Lopes da Silva et al. 2003, where the thalamo-cortical network gives a bifurcation diagram similar to the unfolding of the Bautin singularity (with the presence of subH and FLC bifurcations to create the bistability between the fixed point and the limit cycle); the same bifurcation diagram emerges in the paper mentioned by the reviewer (Suffczynski et al 2004).

This bifurcation diagram (the unfolding of the Bautin singularity) has in fact being used a lot in the context of phenomenological models for seizures, even beyond absence, being present in biophysically inspired models such as the one mentioned by the reviewer (but also Taylor et al. 2011 spatially, Kalitzin et al. 2011 computational, Liley et al. 2001 spatial) and more tractable phenomenological versions of them (such as Kalitzin et al. 2014). The unfolding of the Bautin singularity is included in the DTB model, which can be used to generate phenomenological seizures compatible with both focal and generalized electrographic signatures. This we have discussed in a previous work (Saggio et al. 2020) but we now elaborate in the revised manuscript with these lines:

‘Another advantage of such an approach (*identifying a DTB singularity in the epileptor)* is that it would help identify parameters of the Epileptor model that, if allowed to slowly change, could produce other forms of bursting present in the DTB model, for example those without a DC shift that are absent from the current Epileptor’s map. A lack of DC shift has been observed in some patients with focal seizures, even though this doesn't rule out completely the possibility that the underlying dynamical system is going through a bifurcation exhibiting a jump in the baseline

[Izhikevich, 2000], but the most known type of seizures with this characteristic is ‘absence’. While the Epileptor was designed based on data from focal seizures, the possibility to extend it to generalized seizures in a unifying framework, on the lines of earlier modeling works [Breakspear et al., 2006; Silva et al., 2003], is intriguing.’

III. There different mechanisms modeling the transition to absence seizures were proposed previously
1. change in parameters, like in the early models by Alan Destexhe;
2. transition between two attractors in the bistable (multistable) system; the transition can occur due to an external stimulus from nervus trigeminus like it was proposed in [10.1016/j.neuroscience.2004.03.014] and Ref. [11], or due to some intrinsic processes modelled by noise like in [Taylor P, Wang Y, Goodfellow M, Dauwals J, Moeller F, Stephani U, Baier G. A Computational Study of Stimulus Driven Epileptic Seizure Abatement. Plos ONE. 2014; 9(12): e114316. https://doi.org/10.1371/journal.pone.0114316]
3. seizure together with interseizure interval can be considered as a long lasting event if the model allows this, see [Kolosov AV, Nuidel IV, Yakhno VG. Research of dynamic modes in the mathematical model of elementary thalamocortical cell. Izvestiya VUZ. Applied Nonlinear Dynamics. 2016; 24(5):72-83. doi: 10.18500/0869-6632-2016-24-5-72-83];
4. the epileptic state can be considered as a long transient, see [T. M. Medvedeva, M. V. Sysoeva, A. Lüttjohann, G. van Luijtelaar, I. V. Sysoev. Dynamical mesoscale model of absence seizures in genetic models. PLoS ONE, 2020 15(9), 239125. DOI: 10.1371/journal.pone.0239125] and [N. M. Egorov, V. I. Ponomarenko, I. V. Sysoev, M. V. Sysoeva. Simulation of epileptiform activity using network of neuron-like radio technical oscillators. Technical Physics, 2021 91(3): 519–528. DOI: 10.1134/S1063784221030063].

Nobody knows till now what explanation is right, and some of them could be possible together, but the authors should show that they are familiar with different approaches, what is their own approach and why they chose it.

We completely agree with the reviewer and now include this paragraph in the revision:

‘Once that ultra-slow variables bring the system close to seizure onset, the transition to the ictal state can occur by crossing the bifurcation in parameter space, but also through other mechanisms. These include bifurcations as in the Epileptor, and noise-induced transitions as in the other two big families of models (biophysical or phenomenological) that have been used in the context of large-scale brain modeling for epilepsy, that is bistable models close to a subcritical H bifurcation [Hutchings et al., 2015; Kalitzin et al., 2014; Kramer et al., 2012; Sinha et al., 2016; Taylor and Baier, 2011] and excitable models close to a SNIC bifurcation

[Ermentrout and Kopell, 1986; Goodfellow et al., 2016; Laiou et al., 2019; Lopes et al., 2020; 2019; 2017; Tait et al., 2021; Wendling et al., 2002]. Other mechanisms are possible (see [Saggio and Jirsa, 2022], where we also discuss how fast-slow bursters can accommodate a mixed scenario in which the onset is brought about by noise- induced transitions and, once the system is in the ictal state the slow variable activates to bring the system across seizure offset [Hebbink et al., 2017; Saggio et al., 2020]. This can be achieved by setting the excitability so that the whole system is in a fixed point, as described in Section 4.2.4 and adding noise. Depending on the interplay between the distance to the onset bifurcation and the level of noise, the system can occasionally cross the separatrix (i.e. the middle branch of fixed points) and transition into the limit cycle. It is thus possible to obtain these two onset mechanisms - bifurcations and noise-induced transitions - in the same model, allowing for their coexistence. Analyses of patients' seizures to identify signs of an impending bifurcation have brought mixed results [Scheffer et al., 2009; Wilkat et al., 2019], with the possibility that these onset mechanisms could be patient specific [Maturana et al., 2020], adding another dimension to the taxonomy of seizures based on dynamics.'

Reviewer #2: Compact Bifurcation Analysis of the Epileptor by Maria Luisa Saggio and Viktor K. Jirsa

The MS deals with a key problem of modelling of mesoscopic macroscopic dynamics in the human EEG / MEG in patients with electrographic seizures: it studies and connects transitions between different types of dynamics and thus attempts to explain deterministic components of large scale brain activity. This is crucial for surgical decision making.

A key assumption underlying the work is that seizures result from local dynamical instabilities, typically from a fixed point to some kind of oscillatory behavior. A lot of modelling work rests on purely phenomenological similarity of simulated output with clinical recordings and parameters are empirically fixed to show the desired dynamics but ignoring the potential impact of other parameters. At this point, the MS offers a step forward in reducing the 5-variable epileptor model to the minimal number of variables, which is 3 for bursting, and to thoroughly analyse that minimal model for generic routes in and out of oscillatory behavior (or between two steady states) to understand the more complex 5-variable model with its larger number of parameters.

Overall the MS is of excellent technical quality and clear reasoning.

Specifically, figure 4 gives confidence that the DTB results can be used to predict epileptor dynamics and in particular different routes of transitions and how they relate to each other e.g. given "paths on the map", as the authors show.

Given the newly gained understanding of the dependence of the bifurcation structure on the slowly varying parameters, it will be easier for dynamically trained researchers to adjust the model to observations in time series.

We thank the reviewer for the positive comments, we hope that this manuscript will be a useful tool for researchers wishing to use the Epileptor.

Some comments and critique:

Different phenomenologies are illustrated based on the bifurcations but their relevance to the clinical observations is not discussed. It might good to compare the simulated seizure onset dynamics to the classes proposed by Perucca, Dubeau and Gotman (2013). Particularly, the new classes in Fig. 6.

In our previous work on the identification of dynamotypes in a large-cohort of epileptic patients (Saggio et al. 2020), we have addressed the relationship between dynamotypes and the visual classification proposed by Perucca et al. 2013. We didn’t find any significant relationship between Perucca’s classes and dynamotype, also we didn’t find any apparent relationship between Perucca’s classes and clinical data or pathology. This was true also for dynamotypes, where we didn’t find any relationship with gender, pathology, or localization. We interpreted these results in line with the reply to point 1 from the first reviewer. We found correlations between dynamotype and age, where older patients tended to have more supH offset. A more detailed discussion, if of interest, can be found in Appendix I.9 and I.10. However, the Epileptor model that, in addition to the dynamotype, also encodes an intermediate system for spike and waves, increases the similarity with some of Perucca’s classes, thank you for pointing this out. We now mention this in the manuscript. We added

‘These taxonomies may be orthogonal to other classification systems, pathology or localization [Saggio et al., 2020]. In addition, depending on the focus of a specific investigation, not all

dynamical features identifiable in seizures will carry the same weight. For example, the intermediate subsystem of the Epileptor increases the resemblance between some simulated classes and some seizures observed in patients (as an example, see classes with SupH onset in 6 and Perucca's Low- frequency high-amplitude periodic spikes class (ii)) and is critical for the fast propagation of ictal waves in the form of spike and wave complexes in Epileptor field models [Proix et al., 2018] or to explain synchronous seizure termination across brain regions, but under certain conditions, seems to have a negligible effect when studying seizure propagation across brain regions [Proix et al., 2018]'

One shortcoming of the MS is that while it refers to "seizure", "ictal", and "interictal" states with the clinical application in mind, these are interpretations only. It should state what type of asymptotic dynamics is found (for the analysis, they cut transients away). Are these period, quasiperiodic, chaotic?

We made the fact that these interpretations more evident by writing in section 3.2 in reference to the Epileptor 'This system can display bistability between a stable fixed point (resting state interpreted as interictal condition) and a stable limit cycle (fast oscillatory activity interpreted as the ictal state)'. In section 3.1 about the DTB model instead we kept the more neutral description in terms of fixed points and limit cycles, also using rest and oscillations, and changed in Fig.1 accordingly.

The timeseries we show for bursting, that we 'cutted' for visualization purposes, are periodic solutions, as mentioned in the discussion. We now make it clearer in the results section 4.2.1. 'For visualization purposes, we zoomed in on the seizure in the timeseries. However, these are periodic solutions, with the slow variable pushing the system along the closed paths on the map.'

As with other fast-slow bursting models, both the DTB and the Epileptor model are susceptible to chaotic behaviors for certain choices of the parameters, for example when time-scale separation is greatly reduced (which we prevent) or during the transition from bursting to periodic orbits (as discussed for example in Ermentrout and Terman (2010) for square-wave bursting). For our goals, this may potentially play a role in the transition from seizure to status

epilepticus but we have not investigated this point. We added these lines in the discussion about RSE:

‘In addition, even in the absence of noise, bursting models may display chaotic behavior at the transition from periodic bursting to spiking that may be relevant for RSE [Ermentrout and Terman, 2010].’

A point that might be slightly misleading in the mathematical approach is the implicit assumption that the illustrated dynamics happen in one "location" and thus the choice of parameters determines the "node" dynamics of a lump of e.g. cortex and on top of this connectivity needs to be added to account for spreading. Yet, the dynamics could in principle also be the result of the coupling between potentially distant (in the extreme case trivially behaving) locations. While bifurcations of spatio-temporal models are outside the scope of the MS , it is important that this at least is mentioned.

That is an interesting point that we now mention in the manuscript in section 4.2.4:

‘Even though networks of coupled Epileptors are beyond the scope of this work, we want to point out that Epileptors are usually coupled through a fast-to-slow ’permittivity’ coupling [Proix et al., 2014]. A region’s proneness to seizures can be altered by incoming inputs from other brain regions, while the fast parameters, and thus the class, are unaffected.’

All studies are done in the deterministic case in the absence of noise. While this allows convenient analytic treatments, the corresponding stochastic differential equations might in principle have a more complex dynamics than is expected from the noise-free case. I suggest to point this out and maybe include a citation.

We added these lines in the Discussion ‘A deeper understanding of the transition to RSE in the model with high noise values would require a better characterization of the corresponding stochastic differential equations [Gardiner et al., 1985; Pavliotis, 2016; Risken and Risken, 1996].’

And these in section 6.2.1 : ‘The Epileptor is usually used with low levels of noise that do not alter the dynamics but improve the realism of the simulations. However stronger levels of noise could potentially alter the dynamical repertoire of the model and further analysis is needed to characterize this scenario, especially given the possibility of using the Epileptor with noise-induced transition onsets as described in the Discussion.’

I want to mention that seizure-like dynamics could in principle also originate in behaviors other than passing a slow variable through a bifurcation. E. g. it could be a noise-induced transient exploiting a complicated saddle structure of state space. I leave it to the authors but think it is fair to mention that this specific approach has not yet been conclusively shown to be the correct one.

Please see our reply to comment III from the first reviewer.

Finally, given the good correspondence between the two models, would the authors continue to advise clinical people to work with the complex epileptor rather then with the simpler minimal model? Specifically, the simpler model model might be easier to modify if further variables are added (as per the suggestions in the Discussion).

We think it depends on the goal of the specific research. The DTB can be useful if one is interested in the fast-slow bursting dynamics. But to work well with the additional intermediate subsystem (so to have also the spike and wave complexes and preictal spikes), the parameters should be carefully tuned and we are leaving this for future work.

We have added ‘Future work may address an extension of the Epileptor to these other bursting classes by making the fast subsystem similar to that of the DTB bursting model and carefully tuning the parameters to also guarantee proper coupling with the intermediate subsystem.’

Some remarks:

I think the piecewise model eq (5) is a brilliant tool to work with.

I didn't check the analytical results in 4.1.1

Figure 2 is a nice didactic illustration of the epileptor's working

We thank the reviewer for these positive remarks.

Some suggestions:

line 82: should "deg. DTB" just be "DTB"?

line 382: "iniziation"

line 400: "epileptogenisis"

We fixed these typos.
